# Towards a Functional Cure for Hepatitis B Virus: A 2022 Update on New Antiviral Strategies

**DOI:** 10.3390/v14112404

**Published:** 2022-10-29

**Authors:** Elisabetta Degasperi, Maria Paola Anolli, Pietro Lampertico

**Affiliations:** 1 Division of Gastroenterology and Hepatology, Foundation IRCCS Ca’ Granda Ospedale Maggiore Policlinico, 20122 Milan, Italy; 2 CRC “A. M. and A. Migliavacca” Center for Liver Disease, Department of Pathophysiology and Transplantation, University of Milan, 20122 Milan, Italy

**Keywords:** functional cure, HBV, clinical trials, investigational drugs, nucleos(t)ide analogues

## Abstract

Chronic infection with hepatitis B virus (HBV) represents one of the main causes of the development of cirrhosis and its complications. Treatment with potent third-generation nucleos(t)ide analogues (NUCs) results in >99% HBV DNA undetectability, and prevents fibrosis progression and liver-related complications. However, NUCs are not able to induce the so-called functional cure, which is hepatitis B surface antigen (HBsAg) loss and anti-HBs seroconversion. Consequently, NUC treatment is currently intended as being long-term or lifelong, resulting in the need for clinical monitoring and potentially suffering from compliance issues. Consequently, drug development in HBV has the goal of developing new agents in order to achieve a functional cure for HBV. Currently, the three main strategies include the following: inhibition of viral replication, inhibition of viral antigens, and immune modulation. This review summarizes the most recent updates concerning HBV compounds among these three main classes.

## 1. Introduction

Chronic infection with hepatitis B virus (HBV) affects nearly 300 million people worldwide, representing one of the main causes of the development of cirrhosis and its complications, including hepatocellular carcinoma (HCC) and end-stage liver disease [1,2,3].

Up until today, antiviral treatment with potent third-generation nucleos(t)ide analogues (NUCs) has resulted in effective >99% HBV DNA undetectability with a negligible risk of resistance. Long-term viral suppression prevents liver disease progression and improves liver disease outcomes by reducing the rates of HCC development, decompensation, and the need for liver transplant [4,5,6]. In recent times, the regression of cirrhosis and the improvement of liver fibrosis under prolonged NUC treatment has also been demonstrated [7,8]. As a consequence, long-term HBV DNA suppression represents one of the main treatment goals in chronic HBV infection. NUC-based therapies are easy to administer (oral route) and are free from significant side effects; however, because of their mechanism of action, NUCs rarely achieve the most important HBV treatment goal, which is hepatitis B surface antigen (HBsAg) loss and anti-HBs seroconversion, the so-called functional cure, the only current endpoint allowing for treatment discontinuation. Consequently, NUCs need to be administered long-term, mostly lifelong—such a prolonged treatment can suffer from compliance issues and also requires clinical monitoring for adverse events potentially resulting from some NUC-based therapies (i.e., osteopenia/osteoporosis and kidney disease related to tubular damage) [2]. For these reasons, experimental research into the field of HBV treatment is currently devoted to the identification of new therapeutics targets and drug molecules in order to achieve an HBV functional cure. This review summarizes the most recent updates concerning the HBV compounds under development.

## 2. HBV Life Cycle and New Drug Targets

**Figure 1:**
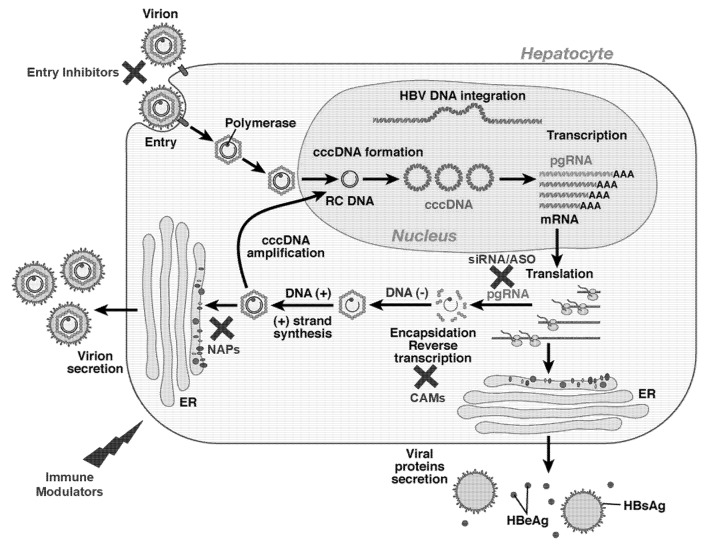
HBV replication cycle and potential antiviral targets. cccDNA: covalently closed circular DNA; RC DNA: relaxed circular DNA; pgRNA: pregenomic RNA; mRNA: messenger RNA; ER: endoplasmic reticulum; HBsAg: Hepatitis B surface antigen; HBeAg: Hepatitis B e antigen; CAMs: capsid assembly modulators; siRNA: small interfering RNA; ASO: antisense nucleotide; NAPs: nucleic acid polymers. Adapted from Zoulim F, et al. Gastroenterology 2009;137:1593-1608 [14].

HBV belongs to the Hepadnaviridae family and is composed of a lipid envelope and a protein capsid containing a partially double-stranded relaxed circular DNA (rcDNA), which is formed by a complete minus strand and an incomplete plus strand [9]. HBV enters the hepatocytes via the Na^+^-taurocholate cotransporting polypeptide (NTCP) receptor; the rcDNA is released and proceeds to the nucleus cell, where it is repaired by the host enzymes and is converted into the covalently closed circular DNA (cccDNA) [10]. Part of HBV DNA can also be integrated into the host genome, which is the main reason for HBV-induced hepatocellular carcinogenesis. cccDNA is a minichromosome that acts as the main HBV replication machinery and whose expression can be modulated through epigenetic changes (i.e., acetylation/deacetylation of histones) [11,12]. Transcription of cccDNA is the origin of several RNAs: pregenomic RNA (pgRNA), pre-core RNA, HBx mRNA, and preS/S mRNA, which serve as templates for the production of all viral particles. pgRNA is the mRNA for the HBV polymerase (Pol) and Core regions, which encodes for viral nucleocapsid (HBcAg) and e antigen (HBeAg). Pre-core RNA controls the translation of the Core region, while HBx mRNA encodes for the HBx protein, a regulatory protein mediating signal transduction, transcriptional activation, and DNA repair, and controlling protein degradation. PreS/S mRNA is the origin of HBs antigens, which includes Large-, Medium-, and Small-HBsAg. After the formation of viral capsid and genome encapsidation, the HBV polymerase (that has reverse-transcriptase activity) starts reverse transcription of pgRNA, resulting in new rcDNA genome formation. The rcDNA-containing nucleocapsid is assembled with all viral proteins, and is enveloped and released. Part of new rcDNA-contaning nucleocapsids can also travel back to the nucleus, where rcDNA is used to replenish the cccDNA pool. This pool is very stable and resistant to degradation, and is depleted only by cell proliferation/elimination and through the help of the immune system. As a consequence, cccDNA represents the basis for HBV persistence and prevents HBV eradication [9,13] (Figure 1).

Current NUCs (inhibitors of HBV polymerase) do not target cccDNA, which decreases long-term only because the inhibition of DNA synthesis reduces the available new nucleocapsids in the nucleus to replenish the cccDNA pool.

While the ideal HBV treatment goal would be the complete elimination of cccDNA (sterilizing cure), compounds to silence/eliminate cccDNA are yet under development and this endpoint will be hard to achieve in the near future. Consequently, the goal of functional cure (HBsAg loss ± anti-HBs seroconversion) is the aim of many investigational drugs under advanced development.

The three main mechanisms of action of new HBV drugs include the following:

1. Inhibition of viral replication
2. Inhibition of viral antigens
3. Immune modulation

While the best antiviral strategy is currently under investigation, evidence is growing that a functional cure could be achieved by a combination strategy of compounds targeting different steps of the HBV lifecycle [13]. As a result, many ongoing clinical trials are investigating the safety and efficacy of drug combinations. An overview of the new HBV compounds under development is provided in Table 1 and Figure 1.

## 3. Inhibitors of Viral Replication—Capsid Assembly Modulators (CAMs)

While the currently available NUCs already inhibit viral replication by targeting HBV polymerase, deeper and more profound inhibition of viral replication could target residual low-level intrahepatic viremia. This drug class includes HBV capsid assembly modulators (CAMs)—they inhibit HBV encapsidation by inducing allosteric conformational changes in the core protein, thus resulting in the formation of non-infectious viral particles (empty nucleocapsids). Because of the generation of aberrant nucleocapsides, as a secondary mechanism of action, CAMs are also supposed to reduce nucleocapsides trafficking to the nucleus and ultimately reduce the cccDNA pool and HBsAg [15].

The addition of 300 mg/day of oral CAM Vebicorvir to the NUC Entecavir (ETV) at 0.5 mg/day for 24 weeks for the treatment of naïve HBeAg positive patients was evaluated in the Phase 2a study 202. Twenty-five patients were included: 68% female, 96% Asian, mean HBV DNA 8.0 LogIU/mL, and mean pgRNA 7.2 LogU/mL. The addition of Vebicorvir to ETV determined a more profound HBV DNA and pgRNA reduction compared with ETV alone, and resulted in 92% ALT normalization at week 24 compared with 17% with ETV alone. Deeper virologic suppression, however, did not result in any HBsAg loss [16].

The CAM RO7049389 was evaluated in combination with NUC ± Pegylated Interferon alpha (PegIFN) in NUC-naïve and NUC-suppressed patients: 72 patients (median age 41 years, 62% males) received either RO7049389 + NUC for 48 weeks (NUC-suppressed patients), or RO7049389 monotherapy for 4 weeks, followed by 44 weeks of RO7049389 + NUC, or RO7049389 + NUC + PegIFN for 48 weeks (NUC-naïve patients). During treatment, a robust HBV DNA decline was observed across all treatment arms, reaching a 7 Log reduction in HBeAg-positive NUC-naïve patients. Five patients met NUC stopping criteria (HBsAg < 100 IU/mL and HBV DNA <20 IU/mL); however, in four out of five cases, relapses occurred. No change in HBsAg was observed, except for patients receiving PegIFN, where aminotransferase (ALT) flares were associated with HBsAg decline [17,18]. CAM ALG-00184 is currently being investigated in a Phase 1b trial in HBeAg-negative and -positive NUC-naïve patients. The administration of ALG-00184 for 28 days at different doses (10, 50, 100, and 500 mg) achieved rapid and profound reductions in HBV DNA and HBV RNA, however without any change in HBsAg levels [19].

The third-generation CAM AB-836 is currently being tested in single or multiple ascending doses in healthy subjects vs. HBV-infected patients for a maximum of 28 days; while complete results are still pending, AB-836 determined a potent inhibition of HBV replication (HBV DNA decline at Day 28 ranging from 3.04 to 3.55 LogIU/mL according to AB-836 dose), however no significant HBsAg decline was reported [20].

Following the negative study results in terms of a decline in HBsAg, a significant CAM interference on cccDNA activity has been questioned. Indeed, the combination of NUC and CAM did intensify the inhibition of replication, i.e., lower levels of HBV DNA and of HBV RNA, compared with NUC alone, but this activity was not associated with HBsAg decline.

## 4. Inhibitors of Viral Antigens

These drugs act by interfering with the different mechanisms responsible for viral entry, the production of viral particles and antigens, and viral assembly. They include the following:

1. Entry inhibitors: they act by blocking the Na^+^-taurocholate co-transporting polypeptide (NTCP) receptor used by HBV to enter hepatocytes.
2. RNA interference: they target viral RNAs to block antigen production. This class includes two different types of compounds:
  - siRNA (small interfering RNA): they act in the cytoplasm by binding to complementary viral mRNAs and triggering their elimination.
  - ASO (antisense oligonucleotide): these modified antisense oligonucleotides target all HBV RNAs to induce the cleavage of HBV RNAs both in the nucleus and cytoplasm via RNase H1.
3. Inhibitors of HBsAg release: nucleic acid polymers (NAPs) act by blocking HBsAg release from hepatocytes and enhancing host-mediated mechanisms of HBV clearance.

### 4.1. Entry Inhibitors

Bulevirtide (Hepcludex^®^, BLV), a first in class entry-inhibitor, is a lipopeptide that mimics the NTCP receptor binding domain, blocking the HDV/HBV entry in liver cells. While BLV received EMA conditional approval in July 2020 for the treatment of compensated chronic hepatitis Delta with an ongoing Phase 3 trial, it is still under investigation in HBV monoinfection. A randomized, open-label multicentre phase 1b/2a trial evaluated daily subcutaneous BLV administration vs. ETV in naïve HBeAg-negative HBV patients. A total of 48 patients (97% Caucasian, 67% males) were randomized to receive different BLV doses (0.5 mg, 1 mg, 2 mg, and 5 mg) for 12 weeks, and either 10 mg/day for 24 weeks or ETV 0.5 mg monotherapy for 24 weeks. A dose-dependent decline in HBV DNA was observed in the BLV arms, although, at best, 75% of patients (vs. 100% with ETV) achieved DNA undetectability, whereas no significant HBsAg declines were observed [21].

### 4.2. RNA Interference

#### 4.2.1. Small Interfering RNAs (siRNA)

The Phase 2b multicenter, randomized, double-blind REEF-1 study investigated the efficacy and safety of the siRNA JNJ-3989 with/without the CAM JNJ-6379 in combination with NUC for 48 weeks in non-cirrhotic (F0-F2) HBV patients. Both HBeAg negative and positive patients NUC-naïve or NUC-treated with HBsAg levels > 100 IU/mL were included: patients were randomized to six arms to receive either NUC monotherapy (Placebo arm), NUC + oral JNJ-6379 250 mg/day, NUC + subcutaneous JNJ-3989 40 mg 100 mg or 200 mg/week, and NUC + JNJ-6379 250 mg/day + JNJ-3989 100 mg/week. After week 48, patients stopped NUC treatment if they met the following discontinuation criteria: ALT < 3 ULN, HBV DNA lower level of quantification (LLOQ: 20 IU/mL), HBeAg negativity and HBsAg < 10 IU/mL. A total of 470 patients were included (median age 43 years, 66% males, 40% Asian ethnicity): patients were stratified according to HBeAg status and history of NUC treatment (naïve vs. NUC-treated at the study start). The primary endpoint of the study was the proportion of patients meeting the NUC stopping criteria at week 48. In the JNJ-3989 arms, HBsAg declined in a dose-dependent manner, especially in HBeAg positive patients (mean HBsAg decline ranging from -2.2 Log in naïve HBeAg-negative patients to -3.6 Log in naïve HBeAg-positive patients), with the HBsAg reduction being >2 Log in 74% and >3 Log in 28% of patients in the JNJ-3989 200 mg/week dose. The primary endpoint (NUC stopping criteria) was met by 19% of patients at week 48 (mostly NUC-treated at baseline) and 30% until follow-up week 24 with the JNJ-3989 200 mg dose. HBV DNA rapidly declined across all treatment arms in NUC-naïve patients, reaching < LLOQ at week 48 in >90% of HBeAg-negative and 50% in HBeAg-positive patients, respectively. A pronounced reduction in Hepatitis B Core Antigen (HBcrAg) and HBV RNA levels were also observed across all treatment populations with JNJ-3989 200 mg/week dose [22].

The Phase 2b REEF-2 study investigated safety and efficacy of JNJ-6379 in combination with JNJ-3989 in NUC-treated HBeAg-negative noncirrhotic (F0-F2) HBV patients. This multicentre European study enrolled 130 patients randomized 2:1 to receive either oral JNJ-6379 250 mg/day plus subcutaneous JNJ-3989 200 mcg every 4 weeks or placebo, in addition to backbone NUC treatment for 48 weeks. As per the study design, at week 48, all treatments (including NUC) were discontinued; the primary endpoint of the study was HBsAg seroclearance (<0.05 IU/mL) at week 72 (+6 months off-therapy). Criteria to restart NUC treatment after treatment discontinuation were HBeAg seroreversion, HBV DNA > 2.000 IU/mL, and ALT > 5 ULN or HBV DNA > 20.000 IU/mL. Among the 85 patients included in the active treatment arm, the median age was 45 years, 68% males, 66% Caucasian under NUC treatment for a median of 8 years. The median quantitative HBsAg was 3.4 LogIU/mL, HBV DNA < LLOQ in 100%, and ALT within the normal range. During active treatment, the nadir of HBsAg decline was achieved between weeks 12–24, and was then stabilized to reach a 1.89 LogIU/mL mean decrease vs. baseline at week 48. At week 72 (6 months off-therapy), none of the patients achieved the primary endpoint (HBsAg < LLOQ without restarting NUC). Nevertheless, 67% of patients had HBsAg levels < 100 IU/mL (vs. 10% in the placebo group), and 30% of patients who had received active treatment maintained HBsAg < 100 IU/mL and HBV DNA < LLOQ (vs. 2% in the placebo arm): 92% of patients had HBV DNA < 2000 IU/mL and 34% < LLOQ, compared with 65% and 10% in the placebo arm, respectively. During the 6-month follow-up after treatment discontinuation, 2% of patients who had received active treatment experienced an ALT flare compared with 24% in the placebo arm, with ALT being <3 ULN in most cases. According to the NUC restart criteria, treatment was re-initiated in 1% vs. 20% of patients who had received active treatment vs. the placebo, respectively. JNJ-6379 and JNJ-3989 were well-tolerated: adverse events leading to premature treatment discontinuation occurred in 3.5% of patients during the active treatment phase. One case of subacute liver failure requiring liver transplantation was reported after NUC discontinuation; this case prompted more stringent NUC restart criteria modification (HBV DNA >20,000 IU/mL, irrespective of the ALT values) [23,24].

AB-729 is a siRNA able to silence all HBV transcripts currently in Phase 2 development: the subcutaneous administration of AB-729 at different doses and schedules (60 or 90 mg; q4, 8 or 12 weeks) for 24–48 weeks was able to induce a mean 1.5 LogIU/mL HBsAg decline that was maintained long-term (up to weeks 80–104). Here, 26/34 patients had HBsAg nadir <100 LogIU/mL and one patient seroconverted HBsAg at week 84. Injection site reactions were the most frequent adverse events, while ALT elevations occurred in 15–43% of cases, which were mostly mild (Grade < 2) [25]

The siRNA VIR-2218 was evaluated in combination with or without PegIFN, either with a 6-dose or 2-dose regimen (200 mg per dose): HBsAg showed a 1.5–2.0 LogIU/mL reduction with a nadir after 16–20 weeks from the treatment start. Grade 1 ALT elevations were reported [26].

#### 4.2.2. Antisense Oligonucleotide (ASO)

The safety and efficacy of the ASO GSK3228836 Bepirovirsen in patients with chronic HBV infection were evaluated in the Phase 2b randomized trial B-CLEAR, where 230 NUC-naïve and 227 NUC-suppressed patients were randomized to four treatment arms: Bepirovirsen 300 mg/week subcutaneously for 24 weeks, Bepirovirsen 300 mg/week for 12 weeks followed by 150 mg/week for an additional 12 weeks, Bepirovirsen 300 mg/week for 12 weeks, followed by 12 weeks placebo, and placebo for 12 weeks followed by Bepirovirsen 300 mg/week for 12 weeks. The primary endpoint was HBsAg < LLOQ (0.05 IU/mL) and HBV DNA < LLOQ (20 IU/mL) 24 weeks after treatment discontinuation; only end-of treatment data (interim analysis) are currently available. Across all treatment arms, patients were >60% males, >50% Asian, 65–76% HBeAg negative, and the median HBsAg was 3.3–3.8 LogIU/mL. During treatment, both NUC-naïve and NUC-treated patients in the 24-week Bepirovirsen 300 mg arms achieved at best 20% and 30% HBsAg <LLOQ at weeks 12 and 24, respectively. Up to 68% in NUC-treated and 65% in NUC-naïve patients achieved HBsAg <100 at the end of treatment, with the virologic response being higher in HBeAg-negative patients with a lower baseline HBsAg (<300 IU/mL). Injection site reactions were the most common adverse events; ALT flares were observed in association with HBsAg decline [27,28]. The occurrence of flares has been linked to the supposed immunomodulatory mechanism of Bepirovirsen, which should stimulate innate immunity by enhancing the TLR pathway in non-hepatocyte cells [29]. Following the positive results of the Phase 2 trial, a Phase 3 study with Bepirovirsen is currently ongoing, while data about the durability of HBsAg loss off-treatment (after Bepirovirsen discontinuation) are expected in the near future from the follow-up period of the Phase 2 trial.

### 4.3. Inhibitors of HBsAg Release

Nucleic acid polymers (NAPs) inhibit the assembly and secretion of HBV subviral particles. The Phase 2 study REP 401 evaluated the safety and efficacy of NAPs REP 2139 or REP 2165 in combination with Tenofovir Disoproxil Fumarate (TDF) and PegIFN in HBeAg-negative noncirrhotic HBV patients. After 24 weeks of TDF treatment, 40 patients were randomized at 1:1 to receive either TDF + PegIFN + intravenous REP 2139 or 2165 250 mg/week for 48 weeks or 24 weeks of TDF + PegIFN, followed by crossover after 48 weeks of TDF + PegIFN + REP 2139 or 2165. The primary outcomes were the safety and tolerability of REP 2139 or REP 2165, while the secondary outcomes were HBsAg decline on treatment and virologic control (HBsAg positive, HBV DNA < 2000 IU/mL, and normal ALT) or functional cure (HBsAg <0.05 IU/mL, HBV DNA < LLOQ, normal ALT) off therapy. Following 48 weeks of TDF + PegIFN + NAP, 24 out of 40 patients achieved HBsAg ≤0.05 IU/mL; off therapy, virologic control persisted in 13 (33%) patients and functional cure was achieved by 14 (35%).

Grade 3–4 ALT elevations were reported in 30 out of 40 (75%) patients receiving the NAP regimen and were correlated with a decline in HBsAg. One participant had a viral rebound during follow-up with hepatic decompensation [30].

## 5. Immune Modulators

One of the mechanisms responsible for HBV infection with chronicization is interference with host-mediated immune responses and T-cell exhaustion. As a consequence, this drug class (whose precursor has been PegIFN) aims at the re-activation of the antiviral immune response and immune stimulation of the natural mechanisms of viral clearance. Differently from IFN, which acted through activating the JAK-STAT pathway and resulted in the transcription of IFN-stimulated genes to induce an “antiviral state” in the host cells, new immune modulators specifically target either innate or cell-mediated immunity. Immune modulators include the following:

1. Toll-like receptor (TLR) agonists: stimulate the TLR pathway to activate innate and adaptive immune responses.
2. Therapeutic vaccines: immunogenic molecules with the goal of generating CD4 and CD8 HBV-specific T cells to eliminate HBV-infected hepatocytes.
3. Immune checkpoint inhibitors: this drug class targets the inhibitors T-cell receptors PD-1 and PD-1 ligand (overexpressed in HBV chronic infection) in order to restore efficacious T-cell responses.
4. Monoclonal antibodies: they bind to specific targets (i.e., parts of HBsAg) to inhibit viral entry, enhance antigen presentation, and stimulate T-cell responses.

### 5.1. Toll-Like Receptors

GS-9620 Vesatolimod, a TLR-7 agonist, was evaluated in a Phase 2 trial: 192 HBV naïve (64% males, 61% HBeAg negative) patients were randomized to receive either different doses of oral Vesatolimod (1, 2, or 4 mg) or placebo once weekly for 12 weeks plus TDF for 48 weeks. The primary endpoint was HBsAg decline at week 24; however, no significant declines in HBsAg levels were observed and none of the patients experienced HBsAg loss. HBV DNA suppression rates were similar across all treatment arms at week 24. A small proportion of patients showed a dose-dependent IFNα induction that was associated with flu-like side effects [31].

The TLR-8 agonist GS-9688 Selgantolimod was investigated in a multicenter Phase 2 study, where 67 HBV patients were randomized to receive oral Selgantolimod 3 mg vs. 2 mg vs. placebo once a week for 24 weeks in combination with Tenofovir Alafenamide (TAF). Patients were 99% of Asian origin, 58% HBeAg positive, 58% men, median age 47 years, HBsAg 4.1 Log IU/mL, and HBV DNA 7.5 Log IU/mL. None of the patients achieved at least 1 Log HBsAg decline at week 24. Nausea, vomiting, and fatigue were the most frequent side effects, mostly of a mild intensity [32]. The 3 mg dose was chosen to be further evaluated in other currently ongoing clinical trials.

### 5.2. Therapeutic Vaccines

The HBV002 Phase 1/2b study investigated the immunogenicity and antiviral activity of VTP-300, a new vaccine with chimpanzee adenoviral vector (ChAdOx1-HBV) and a heterologous modified Ankara boost (MVA-HBV), encoding inactivated Pol, C, and S regions. VTP-300 was administered in combination with a low-dose checkpoint inhibitor Nivolumab at 0.3 mg/Kg intravenously with the aim of boosting VTP-300 immunogenicity. In 55 HBV NUC-suppressed patients, vaccination with VTP-300 combined with low-dose Nivolumab at boost (day 28) was the best strategy, resulting in a >1 Log reduction in four patients and 1 HBsAg loss with sustained responses across the 9-month follow-up period. Treatment was safe and well tolerated, no serious adverse events occurred, most side effects included local injection site reactions, and mild resolving ALT elevation was reported in two cases [33].

The JNJ-0535 HBV therapeutic vaccine (a DNA vaccine encoding Core and Pol administered intramuscularly via electroporation) was tested in HBV patients and healthy volunteers to assess immunogenicity and compare the type and strength of induced immune responses. The administration of JNJ-0535 in a six-dose schedule induced a stronger HBV-specific T-cell response in healthy volunteers than in HBV patients; indeed, 92% of healthy volunteers vs. 50% HBV patients were responders to more than one antigen (Core and/or Pol). The same figures were reported when analyzing the durability of the vaccine-induced immune response, the magnitude of the fold-increase from baseline and the induction of CD4 and/or CD-8 specific T cells [34]. A Phase 1 trial with JNJ-0535 in combination with siRNA JNJ-3989 is currently ongoing.

### 5.3. Immune Checkpoint Inhibitors

A Phase 2b randomized, single-blind, multicenter trial evaluated the efficacy and safety of ASC22 (Envafolimab), a PD-L1 antibody given subcutaneously every 2 weeks. ASC22 was administered in combination with NUC treatment in HBV patients for 24 weeks at two different doses (1 mg/kg vs. 2.5 mg/Kg). Forty-eight patients received the 1.0 mg/Kg dose, median HBsAg 2.9 LogIU/mL, and ALT 22 U/l, all of which were Chinese: three patients with baseline HBsAg <100 IU/mL achieved sustained (week 24 follow-up) HBsAg loss. ALT flares (ALT > 3-fold baseline level) were observed in 21% of patients and were associated with a deeper HBsAg decline. Additional AE included rash and thyroid dysfunction (hypo/hyper) [35]. Nivolumab has been tested in the HBV002 Phase 1/2b study in combination with the vaccine VTP-300 (see Therapeutic vaccines) [33] and is currently being tested in combination with siRNA JNJ-3989 in an ongoing Phase 2 clinical trial.

### 5.4. Monoclonal Antibodies

The Phase 1 study VIR-3434-1002 investigated the safety, tolerability, and antiviral activity of the neutralizing vaccinal monoclonal antibody VIR-3434. VIR-3434 is an engineered human monoclonal antibody targeting the conserved antigenic loop of HBsAg: its mechanisms of actions include the inhibition of viral entry, antigen presentation, and stimulation of T cell, HBsAg clearance, and delivery to dendritic cells. VIR-3434 was administered to non-cirrhotic HBV patients at single ascending doses (6 mg, 18 mg, 75 mg, and 300 mg): treatment was well tolerated and safe, and induced a rapid HBsAg decline (ranging from 1 to 2 LogIU/mL) with a nadir between days 8 and 15, with the 300 mg dose showing the most profound and durable response. At nadir, 23/24 (96%) patients achieved HBsAg <100 IU/mL, while 5/6 (83%) patients receiving the 300 mg dose achieved HBsAg < 10 IU/mL [36].

## 6. Conclusions and Future Perspectives

**Figure 2:**
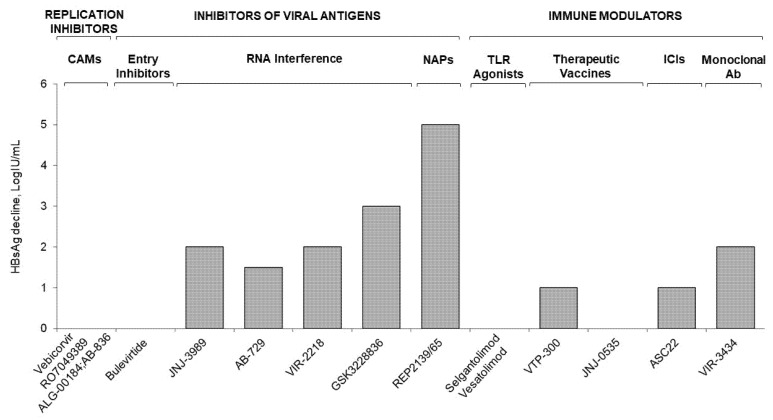
End-of-treatment median HBsAg decline across the main investigational HBV agents.

**Figure 3:**
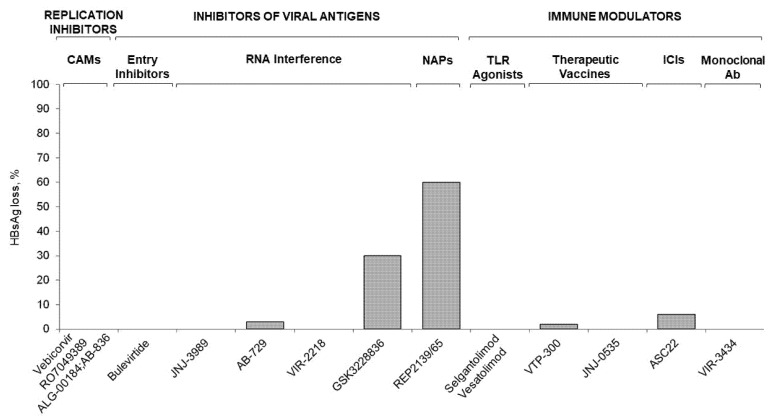
Rates of end-of-treatment HBsAg loss across the main investigational HBV drugs.

The HBV drug development process has resulted in the availability of multiple compounds targeting different steps of the HBV life cycle. An overview of the efficacy results from the main clinical trials is provided in Figure 2 and Figure 3. Despite several studies performed in the last 5 years, not a single therapeutic agent or drug combination has been able to provide significant rates of functional cure in patients with chronic hepatitis B. The search for the most effective and safe therapeutic strategy aimed to achieve a durable off therapy clearance of HBsAg is still underway. Indeed, the profound suppression of viral DNA/RNA by potent replication inhibitors does not reduce HBsAg levels or restore host-specific immune responses. The inhibition of viral antigens achieves a significant on treatment HBsAg decline through RNA interference and up to 30% HBsAg loss with ASO-based regimens. The latter results are associated with ALT flares, as this therapy may restore innate and HBV-specific T-cell responses. However, durability of HBsAg loss off-treatment still has to be assessed, with ASO Bepirovirsen^®^ currently entering a Phase 3 trial. Other inhibitors of viral antigens, such as NAPs, achieve high rates of HBsAg loss and seem to produce durable responses, yet safety issues such as ALT flares have to be addressed. Novel immune modulators (TLR agonist, immune checkpoint inhibitors, therapeutic vaccines, and monoclonal antibodies) achieve variable suppression of HBsAg and restore HBV-specific T cell-responses, but do not achieve sustained HBsAg loss. Consequently, the best strategy for HBV cure will probably be to combine multiple agents in order to target at least two different mechanisms, with the goal to inhibit viral replication, reduce HBsAg burden and restore HBV-specific immune control at the same time.

Currently, the most common strategy adopted by ongoing investigational studies is based on the combination of HBsAg lowering agents (siRNA or ASO) with different types of immune modulators (TLR-agonists, therapeutic vaccines, or immune checkpoint inhibitors). The supposed rationale is that lowering the HBsAg burden would facilitate or restore the capability of the immune system to respond to immune stimulation. For these reasons, the study designs of many Phase 1 or 2 ongoing trials adopt a sequential instead of de novo combination, where the immune stimulation is administered some weeks/months after the siRNA/ASO treatment (always with a backbone NUC). This is the case of ongoing OSPREY Phase 1 trial (siRNA JNJ-3989 followed by addition of JNJ-0535 therapeutic vaccine), ASO-001 Phase 2 trial (Bepirovirsen 12 or 24 weeks followed by chimpanzee adenoviral vector (ChAdOx1-HBV), and a heterologous modified Ankara boost (MVA-HBV)), and OCTOPUS-1 Phase 2 trial (siRNA JNJ-3989 for 16 weeks followed by addition of Nivolumab).

Future studies are needed as well, in order to develop compounds targeting cccDNA to step from the functional cure to the ultimate goal of the complete HBV sterilizing cure.

## Figures and Tables

**Table 1:** Overview of new HBV compounds under development.

Drug Class	Drug Type	Administration Route	Drug Name	Clinical Trials
Inhibitors of viral replication	Capsid assembly modulators (CAMs)	Oral	*JNJ-6379* *ABI-H0731 (Vebicorvir)* *RO7049389* *ALG-000184* *AB-836*	Phase 1/2
Inhibitors of viral antigens	Entry Inhibitors	Subcutaneous	*Bulevirtide (Hepcludex^®^)*	Phase 2
	RNA interference - siRNA (small interfering RNAs) - ASO (antisense oligonucleotide)	Subcutaneous	*JNJ-3989* *AB-729* *VIR-2218* *GSK3228836 (Bepirovirsen^®^)*	Phase 2/3
	Inhibitors of HBsAg release/ Nucleic acid polymers (NAPs)	Intravenous	*REP 2139* *REP 2165*	Phase 1/2
Immune Modulators	Toll-like receptor agonists	Oral	*Vesatolimod* *Selgantolimod*	Phase 2
	Therapeutic vaccines	Intramuscular Subcutaneous Intranasal Intravenous	*JNJ-0535* *VTP-300*	Phase 1/2
	Immune checkpoint inhibitors	Intravenous Subcutaneous	*ASC22 (Envafolimab)* *Nivolumab*	Phase 1/2
	Monoclonal antibodies	Intravenous Subcutaneous	*VIR-3434*	Phase 1

## References

[1] World Health Organization (2021). Global Progress Report on HIV, Viral Hepatitis and Sexually Transmitted Infections. www.who.int/publications/i/item/9789240027077.

[2] European Association for the Study of the Liver (2017). EASL 2017 Clinical Practice Guidelines on the management of hepatitis B virus infection. J. Hepatol..

[3] Raffetti E., Fattovich G., Donato F. (2016). Incidence of hepatocellular carcinoma in untreated subjects with chronic hepatitis B: A systematic review and metaanalysis. Liver Int..

[4] Papatheodoridis G.V., Chan H.L.-Y., Hansen B.E., Janssen H.L., Lampertico P. (2015). Risk of hepatocellular carcinoma in chronic hepatitis B: Assessment and modification with current antiviral therapy. J. Hepatol..

[5] Lampertico P., Invernizzi F., Viganò M., Loglio A., Mangia G., Facchetti F., Primignani M., Jovani M., Iavarone M., Fraquelli M. (2015). The long-term benefits of nucleos(t)ide analogs in compensated HBV cirrhotic patients with no or small esophageal varices: A 12-year prospective cohort study. J. Hepatol..

[6] Su T.-H., Hu T.-H., Chen C.-Y., Huang Y.-H., Chuang W.-L., Lin C.-C., Wang C.-C., Su W.-W., Chen M.-Y., Peng C.-Y. (2016). Four-year entecavir therapy reduces hepatocellular carcinoma, cirrhotic events and mortality in chronic hepatitis B patients. Liver Int..

[7] Chang T.T., Liaw Y.F., Wu S.S., Schiff E., Han K.H., Lai C.L., Safadi R., Lee S.S., Halota W., Goodman Z. (2010). Long-term entecavir therapy results in the reversal of fibrosis/cirrhosis and continued histological improvement in patients with chronic hepatitis B. Hepatology.

[8] Marcellin P., Gane E., Buti M., Afdhal N., Sievert W., Jacobson I.M., Washington M.K., Germanidis G., Flaherty J.F., Schall R.A. (2013). Regression of cirrhosis during treatment with tenofovir disoproxil fumarate for chronic hepatitis B: A 5-year open-label follow-up study. Lancet.

[9] Liang T.J. (2009). Hepatitis B: The virus and disease. Hepatology.

[10] Glebe D., Bremer C.M. (2013). The molecular virology of hepatitis B virus. Semin. Liver Dis..

[11] Nassal M. (2015). HBV cccDNA: Viral persistence reservoir and key obstacle for a cure of chronic hepatitis B. Gut.

[12] Tu T., Budzinska M.A., Shackel N.A., Urban S. (2017). HBV DNA integration: Molecular mechanisms and clinical implications. Viruses.

[13] Loglio A., Viganò M., Lampertico P. (2021). Novel therapies that may cure chronic hepatitis B. Clin. Liver Dis..

[14] Zoulim F., Locarnini S. (2009). Hepatitis B virus resistance to nucleos(t)ide analogues. Gastroenterology.

[15] Zhang X., Cheng J., Ma J., Hu Z., Wu S., Hwang N., Kulp J., Du Y., Guo J.-T., Chang J. (2019). Discovery of novel hepatitis B virus nucleocapsid assembly inhibitors. ACS Infect. Dis..

[16] Sulkowski M.S., Fung S.K., Ma X.L., Nguyen T.T., Schiff E.R., Hann H.-W.L., Dieterich D.T., Nahass R.G., Park J.S., Chen S. (2022). Deeper virologic suppression with the addition of vebicorvir, a first-generation hepatitis B core inhibitor, to entecavir correlates with reduced inflammation and fibrosis-4 index in treatment naïve patients with HBeAg positive chronic hepatitis B. J. Hepatol..

[17] Yuen M.-F., Balabanska R., Hou J., Gane E.J., Lim T.H., Zhang W., Xie Q., Komolmit P., Leerapun A., Yang S.-S. (2022). Viral nucleic acids suppression activity of RO7049389 plus NUC with/without Peg-IFN in virologically-suppressed and naïve chronic hepatitis B patients: 48-week treatment and posttreatment follow-up. J. Hepatol..

[18] Hou J., Gane E.J., Zhang W., Zhang J., Yuen M.-F., Lim T.H., Balabanska R., Xie Q., Komolmit P., Liang X. (2022). Hepatitis B virus antigen reduction effect of RO7049389 plus NUC with/without Peg-IFN in chronic hepatitis B patients. J. Hepatol..

[19] Yuen M.-F., Agarwal K., Gane E.J., Jucov A., Schwabe C., Niu J., Hou J., Le K., Massetto B., Westland C. (2022). Safety, pharmacokinetics, and antiviral activity of the class II capsid assembly modulator ALG-000184 in subjects with chronic hepatitis B. J. Hepatol..

[20] Gane E.J., Jucov A., Kolomiichuk L., Eley T., Brown J., King Y., Medvedeva E., Chernyakhovskyy M., Mani N., Cole A.G. (2022). Safety, tolerability, pharmacokinetics (PK), and antiviral activity of the 3rd generation capsid inhibitor AB-836 in healthy subjects (HS) and subjects with chronic hepatitis B (CHB). J. Hepatol..

[21] Myrcludex B vs. Entecavir in Patients with HBeAg Negative Chronic Hepatitis B. https://clinicaltrials.gov/ct2/show/results/NCT02881008.

[22] Yuen M.-F., Asselah T., Jacobson I.M., Brunetto M., Janssen H., Takehara T., Hou J., Kakuda T., Lambrecht T., Kalmeijer R. (2022). Effects of the siRNA JNJ-3989 and/or the capsid assembly modulator (CAM-N) JNJ-6379 on viral markers of chronic hepatitis B (CHB): Results from the REEF-1 study. J. Hepatol..

[23] Agarwal K., Buti M., van Bommel F., Lampertico P., Janczewska E., Bourliere M., Vanwolleghem T., Lenz O., Verbinnen T., Kakuda T. (2022). Efficacy and safety of finite 48-week treatment with the siRNA JNJ-3989 and the capsid assembly modulator (CAM-N) JNJ-6379 in HBeAg negative virologically suppressed (VS) chronic hepatitis B (CHB) patients: Results from REEF-2 study. J. Hepatol..

[24] Agarwal K., Lok J., Carey I., Shivkar Y., Biermer M., Berg T., Lonjon-Domanec I. (2022). A case of HBV-induced liver failure in the REEF-2 phase II trial: Implications for finite treatment strategies in HBV ‘cure’. J. Hepatol..

[25] Yuen M.-F., Berliba E., Sukeepaisarnjaroen W., Holmes J., Leerapun A., Tangkijvanich P., Strasser S., Jucov A., Gane E.J., Thi E.P. (2022). Long-term suppression maintained after cessation of AB-729 treatment and comparable on-treatment response observed in HBeAg+ subjects. J. Hepatol..

[26] Lim Y.-S., Yuen M.-F., Cloutier D., Thanawala V., Shen L., Gupta S.V., Arizpe A., Cathcart A., Hwang C., Gane E.J. (2022). Longer treatment duration of monthly VIR-2218 results in deeper and more sustained reductions in hepatitis B surface antigen in participants with chronic hepatitis B infection. J. Hepatol..

[27] Yuen R.M.F., Plesniak R., Lim S.G., Tsuji K., Diaconescu G., Gadano A., Kim J.H., Asselah T., Yim H.J., Heo J. (2022). Efficacy and safety of bepirovirsen in patients with chronic hepatitis B virus infection on stable nucleos (t)ide analogue therapy: Interim results from the randomised phase 2b B-Clear study. J. Hepatol..

[28] Lim S.G., Pojoga C., Janssen H., Gusev D., Plesniak R., Tsuji K., Janczewska E., Popescu C.P., Andreone P., Hou J. (2022). Efficacy and safety of bepirovirsen in patients with chronic hepatitis B virus infection not on stable nucleos (t)ide analogue therapy: Interim results from the randomised phase 2b B-Clear study. J. Hepatol..

[29] You S., Delahaye J., Ermler M., Singh J., Jordan W., Ray A., Galwey N., Austin D., Theodore D., Paff M. (2022). Bepirovirsen, antisense oligonucleotide (ASO) against hepatitis B virus (HBV), harbors intrinsic immunostimulatory activity via Toll-like receptor 8 (TLR8) preclinically, correlating with clinical efficacy from the Phase 2a study. J. Hepatol..

[30] Bazinet M., Pântea V., Placinta G., Moscalu I., Cebotarescu V., Cojuhari L., Jimbei P., Iarovoi L., Smesnoi V., Musteata T. (2020). Safety and Efficacy of 48 Weeks REP 2139 or REP 2165, Tenofovir Disoproxil, and Pegylated Interferon Alfa-2a in Patients with Chronic HBV Infection Naïve to Nucleos(t)ide Therapy. Gastroenterology.

[31] Agarwal K., Ahn S.H., Elkhashab M., Lau A.H., Gaggar A., Bulusu A., Tian X., Cathcart A.L., Woo J., Subramanian G.M. (2018). Safety and efficacy of vesatolimod (GS-9620) in patients with chronic hepatitis B who are not currently on antiviral treatment. J. Viral. Hepat..

[32] Janssen H.L., Lim Y., Kim H.J., Tseng C., Coffin C., Elkashab M., Anh S.H., Nguyen A., Chen D., Wallin J. (2021). Safety and efficacy of oral TLR8 agonist selgantolimod in viremic adult patients with chronic hepatitis B. J. Hepatol..

[33] Evans T., Barnes E. (2022). Phase 1b/2a study of heterologous ChAdOx1-HBV/MVA-HBV therapeutic vaccination (VTP-300) combined with low-dose nivolumab (LDN) in virally-suppressed patients with CHB on nucleos (t)ide analogues. J. Hepatol..

[34] de Creus A., Slaets L., Fevery B., van Gulck E., Zhou L., van de Parre T., van den Broeke C., Dimitrova D., Lonjon-Domanec I., Blue D. (2022). Therapeutic vaccine JNJ-0535 induces a strong HBV-specific T-cell response in healthy adults and a modest response in chronic HBV infected patients. J. Hepatol..

[35] Wang G., Cui Y., Xie Y., Mao Q., Xie Q., Gu Y., Chen X.-Y., Hu G., Yang Y., Lu J. (2022). ALT flares were linked to HBsAg reduction, seroclearance and seroconversion: Interim results from a phase IIb study in chronic hepatitis B patients with 24-week treatment of subcutaneous PDL1 Ab ASC22 (Envafolimab) plus nucleos (t)ide analogs. J. Hepatol..

[36] Agarwal K., Yuen M.-F., Wedemeyer H., Cloutier D., Shen L., Arizpe A., Gupta S.V., Fanget M.C., Seu L., Cathcart A. (2022). Dose-dependent durability of hepatitis B surface antigen reductions following administration of a single dose of VIR-3434, a novel neutralizing vaccinal monoclonal antibody. J. Hepatol..

